# X-Band Radar System to Detect Bathymetric Changes at River Mouths during Storm Surges: A Case Study of the Arno River

**DOI:** 10.3390/s22239415

**Published:** 2022-12-02

**Authors:** Francesco Raffa, Ines Alberico, Francesco Serafino

**Affiliations:** 1 Institute of Geosciences and Earth Resources, National Research Council, 56124 Pisa, Italy; 2 Institute of Marine Sciences (ISMAR), CNR, Calata Porta Di Massa—Porto Di Napoli, 80133 Napoli, Italy; 3 Institute of Biometeorology, National Research Council, 57128 Livorno, Italy

**Keywords:** storm surge, X-band coastal radar system, Sentinel 2 image, single-beam survey, flooding event, river mouth, sediments

## Abstract

Storm surges are natural events that influence the dispersion of sediment along coasts, leading to sudden morphological changes in the seabed. From this perspective, we focused our study on the analysis of measurements from a mobile X-band radar system to survey the sea state and the changes in the seabed depth during storm surges. This analysis was supported by additional information from Sentinel 2 satellite images, the *Gorgona wave buoy*, the *San Giovanni alla Vena* hydrometric station, and an echosounder survey. The survey period was from 26 to 28 February and 3 March 2020. During these days, the simultaneous occurrence of a storm surge and flooding of the *Arno River* was monitored. The analysis of the marine X-band radar mobile images determined the formation and dismantling of seabed shapes. An elongated shoal and a bar-like shape are visible on the right side of the *Arno River* in the radar image of 26 February and at the *Arno* mouth on that of 28 February, respectively. The radar image of 3 March shows, near the mouth of the Arno, a delta shape probably due to the deposition of sediment favoured by the interaction between the river flow and storm waves. X-band coastal radar is a detection system that improves the effectiveness and reliability of coastal monitoring because it has a high temporal and spatial resolution. It can be considered a valuable warning system to monitor the sea-bed depth changes in strategic sites, such as harbour areas, during sea storms. Moreover, this system, together with a satellite observing system, is a valid tool for shedding light on the environmental drivers that reshape coastal areas.

## 1. Introduction

In recent centuries, coastal areas have undergone significant changes driven by both anthropogenic and natural factors. The presence of coastal defence structures, ports, river dams, sediment mining in rivers, as well as coastal subsidence and flooding events, strongly influence the erosion tendency of coastal areas. Furthermore, the effects of coastal erosion are exacerbated by climate change. The relative sea level rise and increased storm surges are some of the main drivers of high rates of coastal erosion [1]. The constant action of waves, wind, and currents as well as seasonal changes in the surf zone between storms and calm periods contribute to small-scale changes in beach morphology in a time interval varying from hours to years ([2] and references therein). River floods also belong to this category of events. Sediments deposited by rivers cause changes to coastlines for a few months, while large floods change their shape for years to decades [3,4,5]. In addition, urban areas represent an element of instability that prevents the coastal system from adapting naturally to new environmental conditions, and at the same time, they represent vulnerable areas increasingly exposed to natural hazards ([6,7] and references therein).

Morphological changes in coastal areas, exposed to natural and anthropogenic forcings, have been extensively studied and well documented [8,9,10,11,12,13]. Several studies have focused both on the response of coastal areas to storms [14,15,16] and on the damage suffered by the populations and biota [17,18,19]. The phenomenon of coastal erosion has devastating consequences, such that it threatens the preservation of coastal cities, in terms of the loss of homes and lives and the destruction of natural habitats for animal and plant species. In addition, coastal erosion greatly decreases the value of beachfront property and the value of real estate, due to the fact that the beach is no longer able to accommodate boating, recreational, and fishing activities [20].

X-band radar systems have been used in coastal monitoring to detect morphological changes at a river mouth [21,22,23,24]. Remote sensing techniques are becoming increasingly popular thanks to their many advantages over in situ methods [25,26,27,28]. X-band radar systems, or Wave Radars, have established themselves among the monitoring systems thanks to their operational ease. Standard nautical Wave Radar systems, using radio waves of 9 GHz, make it possible to scan the sea surface with a high temporal and spatial resolution; therefore, they are able to monitor the sea state in time and space based on the techniques pioneered by [29]. There are several approaches to extracting wave and current statistics [30,31,32,33,34,35,36,37]. In this respect, the processing of data from the echo reflected by the sea surface state makes it possible to obtain essential information, such as wavelength, direction, and period of dominant waves, surface currents, and bathymetry of the seabed [38,39,40,41,42]. In addition, Wave Radars offer advanced operational flexibility due to their small size, light weight, and easy installation, although the cost may be prohibitive for developing countries.

This paper expands on the previous research and demonstrates that Wave Radars have a key role in monitoring sea state and seabed depth changes during storm surges in nearshore areas such as river mouths, harbours, and other strategic sites. The study area was the coastal zone close to the mouth of the *Arno River*(Tuscany Region, Central Italy) because it is exposed to storm surges and flooding events and, therefore, provides the possibility to monitor these events and their interaction. Furthermore, the dense urbanization extending to the coastline makes this area very vulnerable to natural hazards.

In the present work, Wave Radar data provided a suitable dataset to monitor the sea state and derive the seabed morphology at the *Arno River* mouth during the storm surges that occurred between 26 and 28 February and on 3 March 2020, when a river flooding also occurred. We also analysed the data registered by (i) the *Gorgona* wave buoy (https://www.sir.toscana.it/mareografia-pub accessed on 9 June 2020) to point out the frequency of storm surges with significant wave height; (ii) the hydrometric station of *San Giovanni alla Vena*, 31 km from the *Arno* mouth (https://www.sir.toscana.it/idrometria-pub accessed on 10 June 2020), to identify the recurrence of flooding events; and (iii) the echosounder survey conducted on 10 September 2020 in calm conditions to detect the morphology changes of the seabed. Furthermore, the analyses of Sentinel 2 level 2 images made it possible to observe the dispersion of the river plumes.

## 2. Study Area

**Figure 1:**
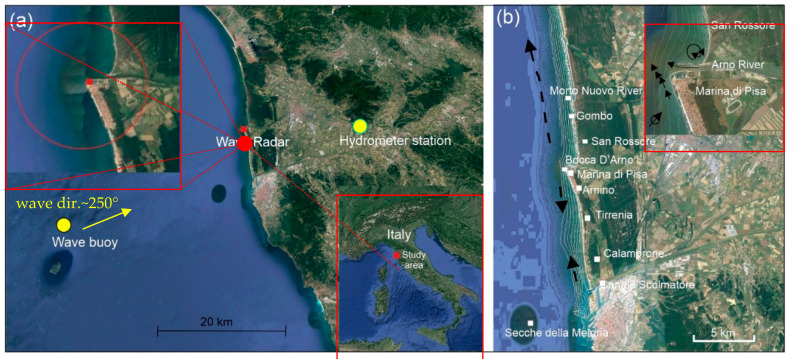
(**a**). The wave buoy of “*Gorgona*”, with a close arrow to indicate the dominant direction of storm waves observed during the present survey, and the hydrometer station of “*San Giovanni alla Vena*” are shown. The surveyed area is highlighted with a red circle in the frame on the upper left while the study area is reported in the frame on the lower right. (**b**). The main direction of longshore current (black arrows) and a detail at *Arno* mouth, in the upper right of image, are shown.

The *Arno River*, 241 km long, is the main river in Tuscany (Central Italy). It has a basin of around 8200 km^2^ and it flows into the Tyrrhenian Sea at Marina di Pisa (Figure 1a).

At present, the *Arno River* delta, stretching out to sea, breaks the linearity of the coastline and favours a divergence in the seawater circulation (Figure 1b). The sediment load amounts to approximately 1524.000 t/yr [43], feeding both sides of the coast reaching the localities Calambrone southward and Gombo northward, as defined by the grain size and petrographic analysis of the sediments that form the beaches [44]. An emerging mouth bar, indicating a significant sediment discharge from the *Arno River*, was originally noted via its first representation by Leonardo da Vinci in 1503 [45]. In 1606, the shape of the *Arno* mouth changed greatly after its northward 1.547 m displacement [46]: this was decided in order to reduce the land inundation, since the new position was less exposed to the prevailing southwesterly wind (Libeccio), which hindered the free discharge of the river flow into the sea [47].

The *Arno River* course was again significantly modified in 1771. The bend immediately below the city of Pisa, an area frequently subjected to flooding, was eliminated by straightening the river’s path. As a result, the river load flowed directly into the sea, and progradation of the coast occurred over several kilometers, as evidenced by the curved shape of the beach ridges and a huge delta cusp at the river mouth [47,48,49]. In the late 18th century, the progradation decelerated and was replaced by coastal retreat [46,50], mainly due to anthropic activities that caused a reduction in the sediment supplied by the river [46,48]. The delta of the *Arno River* developed asymmetrically [45] since its left side, the location of the Marina di Pisa settlement, was protected with defence works in around 1872, while the right side, as a natural area, was exposed to intense erosion processes and a coastline retreat totalling more than 1 km versus the 300 m on the left side [48]. In the mid-1960s, ten groynes were built close to the *Arno River* mouth, and five detached breakwaters were situated further to the north near Gombo [51] in order to prevent further retreats. This intervention produced only a temporary positive effect—a tombolo-like shape that was successively eroded together with the downdrift sector of the beach [52,53]. In 2009, other defence works (geotube, groynes) were placed in this area.

At present, the sand removed from the southern sector is transported northward, according to the littoral drift, up to the southern pier of the Morto Nuovo River mouth. This latter structure determines an updrift deposition of sediments that promotes an increased beach width but, at the same time, a loss of sediment in the downdrift zone [54].

The left side of the *Arno mouth* is exposed to intense erosion, the sediment of which has partially fed the southern sector, which has experienced accretion or equilibrium phases [54,55]. The sea walls, detached breakwaters, and groynes built at Marina di Pisa since the early 20th century have created around ten cells to ensure a stable coastline position; conversely, the wave reflection caused by breakwaters has determined a steady deepening of the sea floor in front of the structures, which requires continuous replenishing. The sandy beach environment at Marina di Pisa has been replaced by artificial coarse-clastic beaches made of marble pebbles [45,56], pointing to the groynes as a cause of the accelerated coastal erosion because, during sea storms, the sea waves channelled between two solid structures accelerate and reach the beach with high energy. In addition, the energy of river floods, no longer reduced by territorial inundation, erodes the riverbed, as was the case during the flood events of 1844 and 1920. The Arno River flow at the S. Giovanni alla Vena station, where the river received runoff from all its tributaries, has an average flow of 90 m^3^/s, a minimum flow of 2.2 m^3^/s, and a maximum flow of 2.250 m^3^/s [57]. Flow rates with higher values occur during exceptional events, such as that of 4100 m^3^/s recorded during the flood event of 4 November 1966 [58]. This area is also exposed to sea storms. About 90% of the events that occur every year along the coast of the province of Pisa (average value = 48 events) come from the 220°–260° sector [59] according to the dominant wind regime belonging to quadrant III [60].

Along the Mediterranean coasts, tides have an average tidal amplitude of about 40 cm, with the exemption of outstanding tides observed in the Gulf of Gabes and in parts of the North Adriatic sea, where they may reach amplitudes up to 1.80 m [61].

## 3. Materials and Methods

The wave climate and seabed morphology were defined on the analysis of Wave Radar images. The results of wave climate analysis were validated with sea state data registered from the Gorgona buoy off the Arno River, while the seabed morphology echosounder was used to define the accuracy of the seabed morphology. Furthermore, Sentinel 2 level 2 satellite images from 2015 (data for which images are available) to 2022 were analysed to deduce the possible direction of the near-shore sea current from observations of river plume dispersion during concurrent flooding and storm events.

The single dataset and the procedures applied to integrate the information suitable for the present study are described in the following sub-paragraphs.

### 3.1. Wave Radar Systems

**Figure 2:**
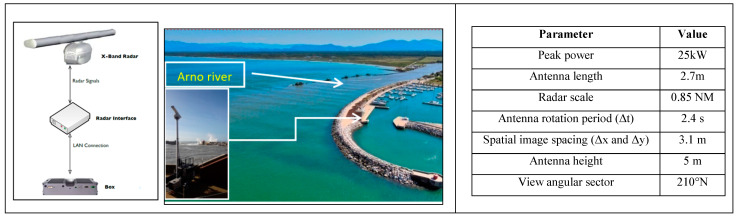
Wave Radar system: hardware architecture on the left. In the middle: photo of the installation site at the port of Marina di Pisa. On the right are the listed Radar system parameters.

Wave Radar data were continuously acquired by the Mobile Remocean Coastal Monitoring system, deploying a Consilium/Selesmar marine X-band radar with a power of 25 KW, operating in the short pulse mode (i.e., pulse duration of approximately 50 ns) and an antenna 9 ft (2.7 m) long with horizontal polarization (HH) (Figure 2). The Mobile Remocean Coastal Monitoring system, located near the control tower of the port of Marina di Pisa (coordinates: Lat. 43°40′37″ N and Long. 10°16′08″ E), analysed the raw data sequences of 128 consecutive images at 2.4 s. intervals between 26–28 February and 3 March 2020.

The Wave Radar system, with each radar antenna revolution, scans the sea surface with a high spatial and temporal resolution; this is achieved by the interaction (backscattering) of the electromagnetic waves (microwaves) emitted by the radar with the capillary waves (ripples) generated by the wind on the sea surface. The backscatter intensity received from the sea surface is transferred via an isolated buffer to the AD converter in a PC. The intensity of the reflected radar signal decreases with the fourth power of the distance. Currently, the analysis is restricted to an area of a few kilometres from the radar; in fact, at greater distances, due to the attenuation of the signal received, the backscattered energy input from the sea becomes more like noise than a signal. In the Wave Radar system, the data are stored, and wave analysis is conducted. One Wave Radar measurement is based on the analysis of several radar images (default is 32 images). The resulting wave information, including individual wave spectra and a time series of the main statistical wave parameters, are stored and displayed on the Wave Radar graphical user interface (GUI) [62,63].

**Figure 3:**
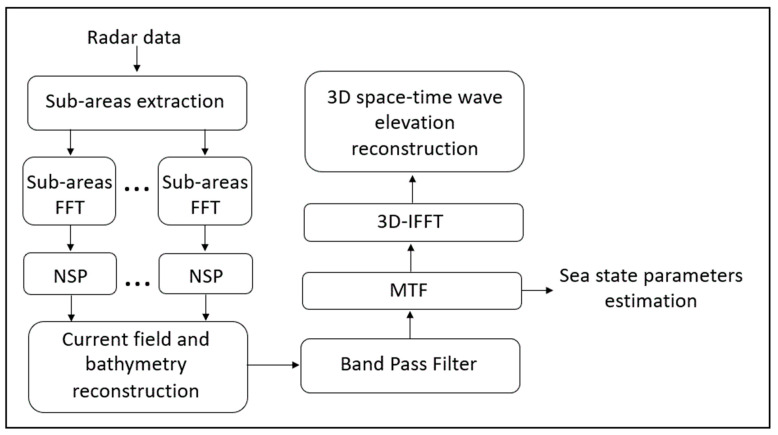
Block diagram of the inverse procedure for radar data analysis.

Longer gravity waves are visible in the radar images as they modulate the backscattered signal generated by capillary waves [63,64,65]. It must be stressed that the images from the radar—due to the particular acquisition geometry that causes distorting phenomena, known as modulation phenomena—are not the direct representation of the sea surface; therefore, a data processing procedure must be introduced to reduce the distortion effects. From a computational standpoint, data processing consists of solving an inverse linear problem which, starting from a series of consecutive radar images, provides the space–time wave elevation. The inversion scheme requires several steps (Figure 3).

The first step of the algorithm is to apply the 3D fast Fourier transform to the raw radar data images. Due to the proximity of the coast, the hypotheses of homogeneity and uniformity of the data cannot be considered valid [36,65]. For this reason, the normalized scalar product (NSP) technique [37,66] procedure was applied to sub-areas to estimate the current field and bathymetry by means of the dispersion relationship:

$$\omega =\sqrt{gk\;\tanh\left(kh\right)}+\bar{k\;}\cdot \;\bar{U}$$

where ω is the angular frequency, g is the acceleration due to gravity, $\bar{k}=\left(k_{x},\;\;k_{y}\right)$ is the wave number vector, and $\bar{U}=\left(U_{x},\;\;U_{y}\right)$ is the surface current vector, and h is the depth of the sea.

Both the surface currents and the bathymetry may be estimated by estimating the maximization of the following normalized scalar product:

$$V^{j}=\left(\bar{U},h\right)\frac{\left|F_{I}^{j}\left(\bar{k},\omega \right)\right|,G^{j}\left(\bar{k},\omega ,\bar{U},\;h\right)}{\sqrt{P_{F^{j}}}\cdot P_{G^{j}}}$$

where $\left|F_{I}^{j}\left(\bar{k},\omega \right)\right|$ is the power spectrum in the *j*th patch, $G^{j}\left(\bar{k},\omega ,\bar{U},\;h\right)$ is the characteristic function built by the mean of the dispersion relation above, and $P_{F^{j}}$ and $P_{G^{j}}$ are the radar power spectra $\left|{F_{I}}^{j}(\bar{k},\omega )\right|$ and $G^{j}\left(\bar{k},\omega ,\bar{U},\;h\right)$, respectively.

Once the current field and bathymetry maps have been estimated, a bandpass (BP) filter may be generated by exploiting the dispersion relationship to delete the undesired spectral components induced by the modulation effects [30,36,64,66]. To compensate for the radar spectral distortion due to the specific radar geometry and to transform the radar spectrum into the sea spectrum, the radar spectrum is multiplied by a modulation transfer function (MTF), from which it is possible to extract the sea state parameters. The final step is to apply the 3D Fourier inverse transform to the sea spectrum to return to the space–time domain, obtaining the evolution of the wave elevation η (x,y,t) [67].

The depth estimation procedure determines the depth of the seafloor that better “correlates” the measured sea-wave spectrum and the characteristic function as the locus of points in the dispersion relation evaluated for different values of sea depth. In general, this method is used to determine both the surface currents and the sea depth. All bathymetry estimates were obtained as time averages of eight daily consecutive measurements.

In this case study, the low amplitudes of the tides in this part of the Mediterranean indicate a weak influence of the tides in a storm situation. It must be mentioned that the moon phase was crescent from 26 of February toward the first quarter on the 2 of March. When the storm event occurred, the mean tide range was 14.2 cm; therefore, the tides could certainly be considered negligible for the determination and analysis of the bathymetry.

### 3.2. Sea State Parameters Measurements

Three miles off the island of *Gorgona*, 42 km from the coast opposite Marina di Pisa, a system for monitoring the wave motion, consisting of a wave buoy and a data reception system, was installed by the Hydrological Service of Tuscany (SIR). The buoy is anchored in the seabed at a depth of around 140 m, and it is able to provide the wave height, direction, period, and energy spectrum parameters, which are sent to a land station by radio or, alternatively, by an IRIDIUM satellite transmission. These data, with half-hour steps, can be downloaded from the “Mareography” page of the Tuscany Region website (https://www.sir.toscana.it/mareografia-pub, accessed on 9 June 2020).

For the study area, we analysed the data recorded by this buoy, which can be freely downloaded from the website of SIR (https://www.sir.toscana.it/mareografia-pub, accessed on 9 June 2020) where both historical and daily data are available. The wave measurements collected during the sea storm, analysed in the present work, were used in order to validate the wave data derived from the Remocean radar system.

### 3.3. Echosounder Survey

The Garmin model 8410XSV combo chartplotter/echosounder was used to define the accuracy of the seabed morphology defined by marine X-band radar. This instrument, with a dual operating frequency of 70/200 kHz, can survey, in a single-beam mode, the seabed at a depth of up to 120 m. The depth of the seabed was measured with a 6-meter inflatable boat at points distributed in a regular grid that extends from the coast to 1000 m offshore. The measurement was processed to generate the digital model of the seabed. Data georeferencing was ensured by the Hemisphere Vector V103 Compass GPS, consisting of a double antenna that provides positioning data but also acts as a gyrocompass. For the storm surge analysed in the present work, it was not possible to measure the accuracy of the seabed morphology defined from X-band radar images because of the lack of seabed morphology pre-events. The accuracy of the seabed depth measurements was defined for a storm surge that occurred on 10 September 2020 by comparing radar measurements with those acquired with the Garmin model 8410XSV combo chartplotter/echosounder, immediately after this event.

### 3.4. Hydrometer Measurements

The data recorded at the “S. Giovanni alla Vena” station (coordinates: Lat. 43°41′04.51″ and Long. 10°35′07.22″), the closest to the coast, located at 31 km inland from the mouth of the *Arno River*, were analysed for this work. In spite of the distance from the mouth, this station is reliable for the calculation of the river flow rate because seaward from it, the contributions of new stream waters can be considered negligible with respect to the rainfall collected from the basin. The data were validated by the periodic measurements at the river mouth taken by the SIR (https://www.sir.toscana.it/idrometria-pub, accessed on 9 June 2020). The hydrometric data from the 2015 to 2022 time windows were downloaded from the SIR to identify significant flows of the Arno River during storms.

### 3.5. Satellite Images

The Sentinel 2 satellite images provided data in 13 spectral bands ranging from visible and near-infrared to short-wave infrared (443–2190 nm) regions and have a spatial resolution of 10 m for visible bands used for the present work. Eight images, from 2016 to 2022, were downloaded from the Copernicus site (https://sentinels.copernicus.eu/web/sentinel/sentinel-data-access, accessed on 2 October 2021). For each image, the plume dispersion was visually analysed to identify its main direction [68] during the concurrence of flooding events and storm surges. The analysis of the spread of the river plumes was beyond the goals of the present work.

## 4. Data Analysis

**Figure 4:**
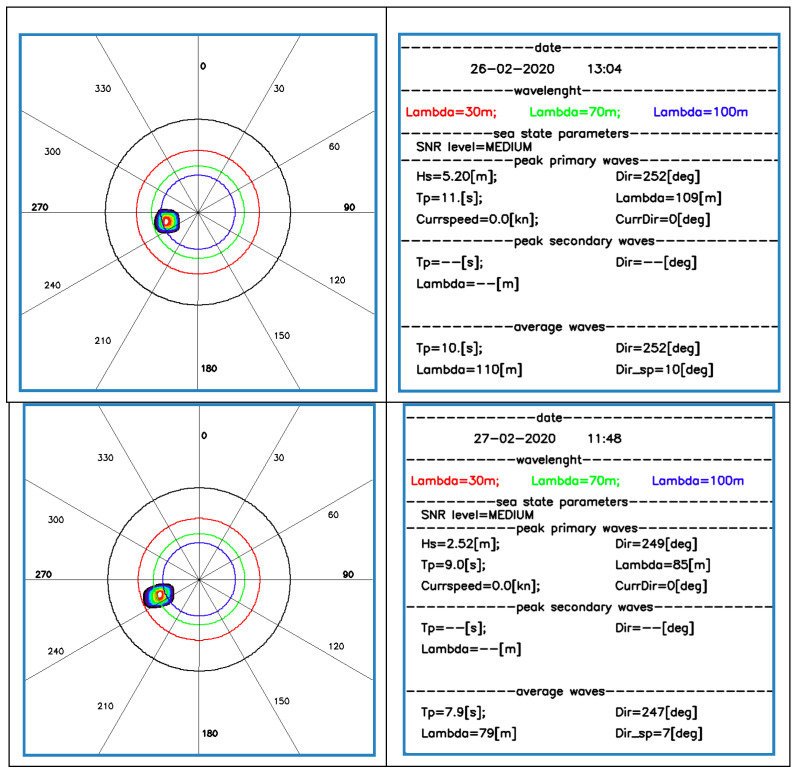

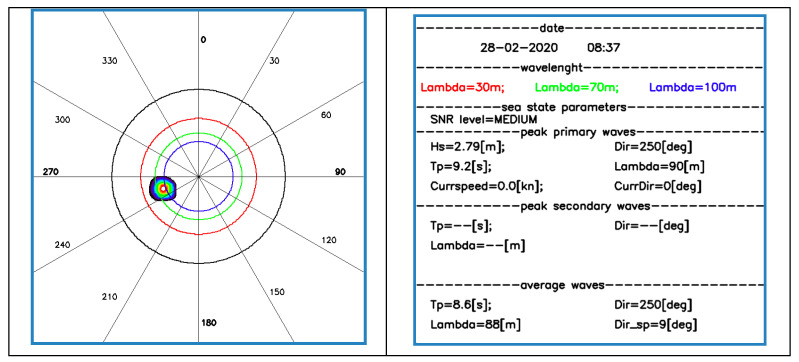
Directional spectra (on the **left**) and wave parameter measurements (on the **right**) from 26 to 28 February 2020.

The Wave Radar data, in real time and with high accuracy, were able to provide several characteristic parameters of the sea state: the significant wave height (Hs), the direction (Dir), period (Tp), and length of the dominant waves (lambda) (Figure 4). For all monitoring days, 26–28 February and 3 March, the storm waves came from 220°–260°, which was the dominant wave direction for the study area [61].

**Figure 5:**
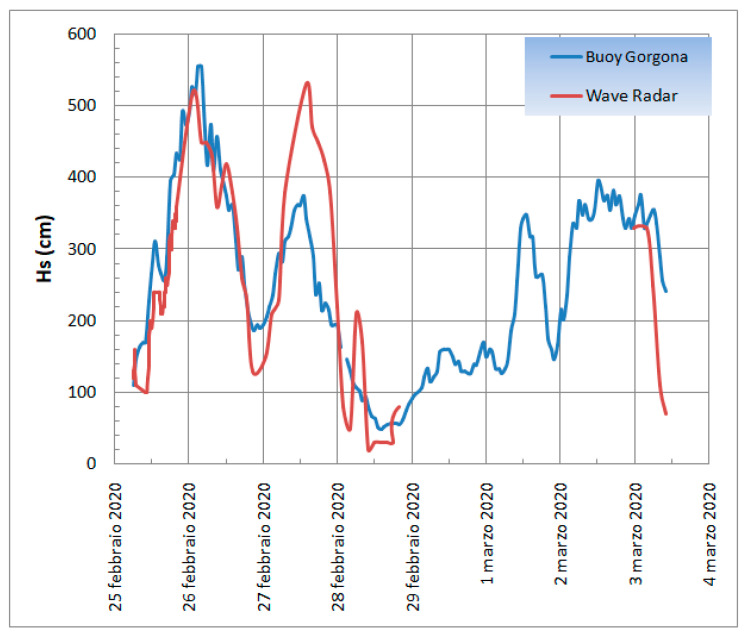
Mean wave height measurements recorded by the *Gorgona* wave buoy (blue) and Wave Radar (red).

The analysis of Hs recorded by Wave Radar from 25 February to 4 March 2020 shows three peaks with a wave height of about 6 m on 26 February and 4 m on 28 February and 3 March (Figure 5). The peaks are also evident in the dataset of significant wave heights recorded by the *Gorgona* buoy station (Figure 5). The data comparison shows a similar trend for both datasets. Given the minimal tidal ranges, the tides are again negligible in this comparison.

The radar system also made it possible to reconstruct the morphology of the seabed near the mouth of the *Arno River* within a radius of approximately 1.6 km from the in-station radar site and in the bathymetric range between 6 and 14 m. In the investigated time window, the depth varied between 7.5 and 12 m and showed the formation of several morphological changes in the seabed.

**Figure 6:**
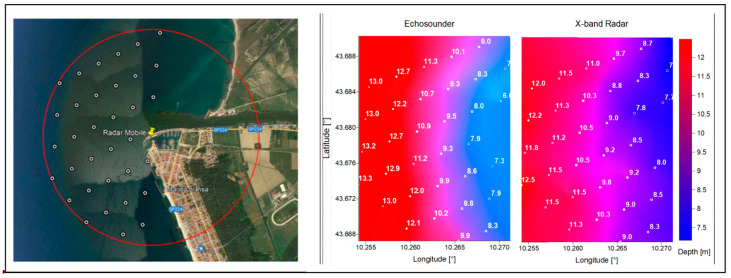
Comparison of bathymetry measurements: the grid points in the survey on the (**left**), the bathymetry obtained by the echo-sounder in the middle, and by Wave Radar measurements on the (**right**).

The reliability of the seabed depth surveyed from marine X-band radar was tested on a storm surge that occurred on 10 September for which a grid derived from the echo-sounder data, surveyed immediately after the storm surge, is available. A comparison of grids derived from these two datasets shows that both images are characterized by a gradual deepening of the seabed, except a more regular shape is visible at the colour change in the echosounder map (Figure 6).

**Figure 7:**
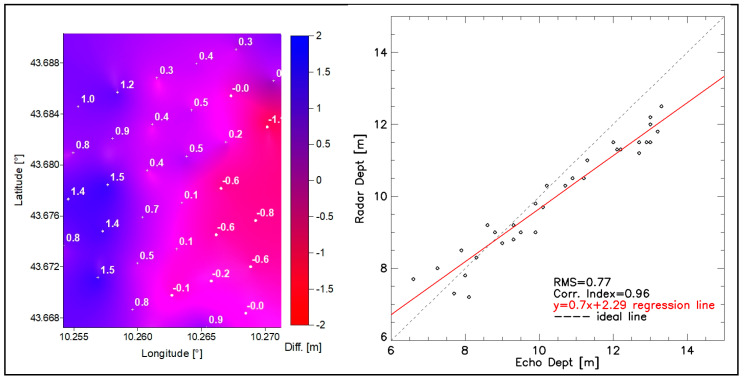
Map of the differences between the bathymetry measurements made with the echosounder and Wave Radar (**left**); scatterplot with regression line (**right**) and correlation index.

To validate the reliability of the seabed morphology derived from the Wave Radar survey, this visual analysis was also corroborated by a map of the differences between the two different methods of bathymetry measurements, and then by a regression analysis between the depth values of the echosounder and the radar. The analysis obtained a significant correlation index of 0.96 and a root mean square (RMS) error of 0.77 m (Figure 7).

**Figure 8:**
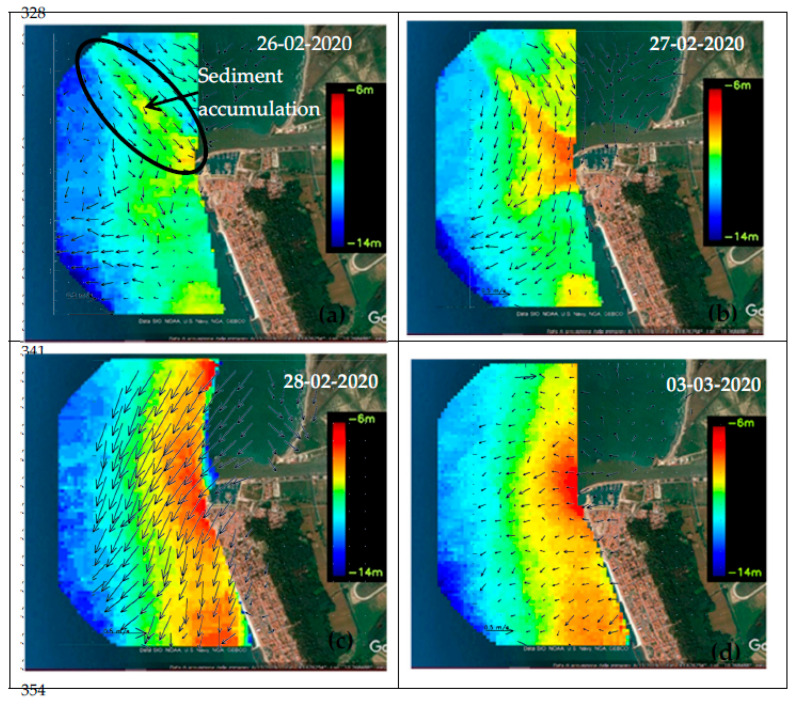
Surface currents overlaid to the bathymetry maps of 26 February (**a**), 27 February (**b**)28 February (**c**) and 3 March (**d**), 2020, as surveyed and processed by the Remocean Coastal Monitoring system.

Wave Radar images from 26 to 28 February and 3 March 2020 show the changes in the depth of the seabed (Figure 8a,d).

On 26 February at 4:37 p.m., the significant wave height (Hs, the statistical parameter normally used to measure sea state) was about 5.5 m. The seabed depth map shows an elongated shoal with a depth of about 8–9 m at the river mouth (Figure 8a). This shape was mainly due to both the high energy of the storm surges, which eroded the sediments from the seabed and transported them landwards, and to the high energy of the longshore current, which moved sediments mainly northwards.

On 27 February at 00:49 a.m., the sea waves were characterized by low energy (Hs: 2–3 m).

The seabed depth map shows an area with a bifurcated shape at the river mouth that was characterized by the highest sediment accumulation of the surrounding area as evidenced by its shallower depth (orange-red colours, Figure 8b). This was due to the lower energy of the swells and of the longshore current that favoured the accumulation of sediments eroded from the seabed on both sides of the *Arno* mouth.

On 28 February at 08:37, the coastal system was affected by sea waves with higher energy (Hs: 4m), and the sediments continued to accumulate in front of the *Arno* mouth in a bar-like structure parallel to the coast, with a depth of about 6 m b.s.l. (Figure 8c). The shallower area of the sea depth (red colour) in the lower part of the images, already visible in yellow on the map of 27 February, was an accumulation of sediments from the *Scolmatore channel* (Figure 1), as highlighted by the concentration of suspended particulate matter (SPM), drawing a plume diverging northward, in the Sentinel 2 satellite images reported below.

**Figure 9:**
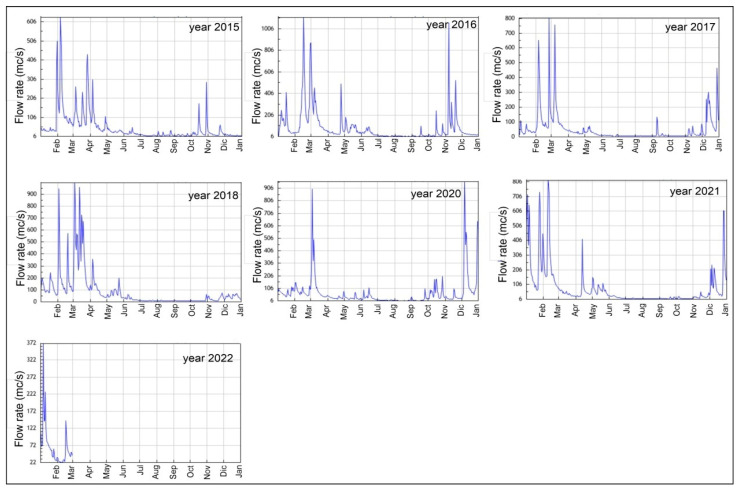
Flow rate of the *Arno River* between the years 2015 and 2022.

On 3 March 2020, there was a flooding event that occurred simultaneously with a storm surge that was slightly weaker than the previous one (Hs: 3 m). This condition made it possible to study the effect of the interaction between these two events on the seabed depth. On this day, at the *S. Giovanni alla Vena* hydrometric station, a river flow rate of about 950 m^3^/s, a typical value for the October–January time window, was recorded (Figure 9).

To support our analysis onthe dispersion of the *Arno River* plume mainly northward, we analysed the Sentinel 2 satellite images (https://sentinels.copernicus.eu/web/sentinel/sentinel-data-access, accessed on 30 July 2022) from the months of October to March, typically characterized by storm surges and high average daily flow rates of the *Arno River* (Table 1).

**Figure 10:**
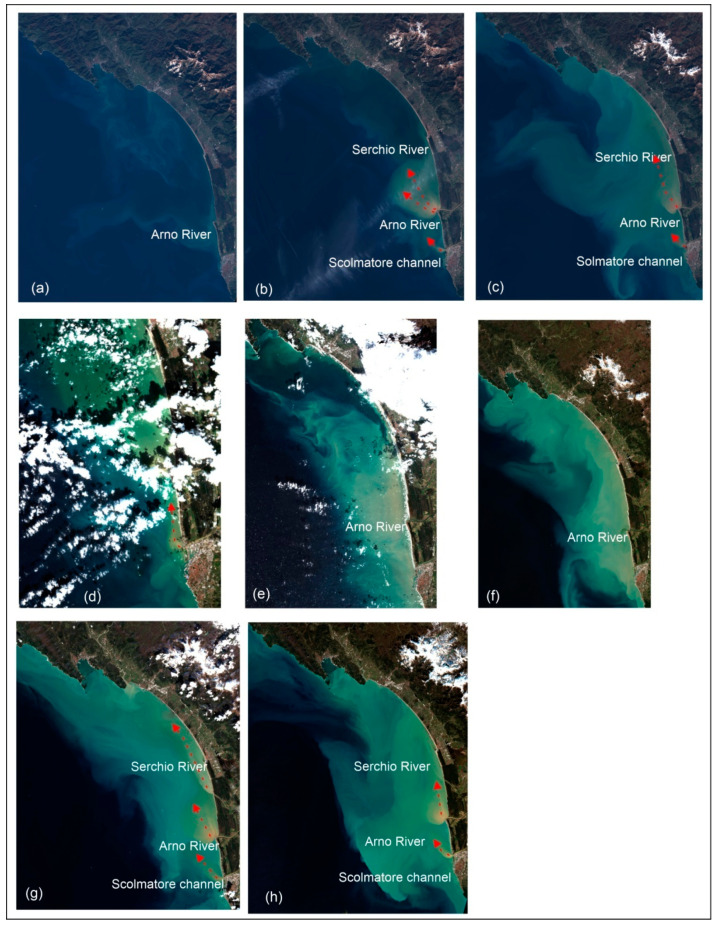
The Sentinel 2 images show the plumes of the Arno River, Serchio River and Scolmatore channel during daily condition (**a**) and flooding events occurred on 26 February 2017 (**b**), 8 March 2017 (**c**), 8 March 2018 (**d**), 26 February, 2020 (**e**) 28 February 2020 (**f**), 4 March 2020 (**g**), 26 January 2021 (**h**). The red arrows indicate the main direction of plume dispersion.

We found seven images with evident plume dispersion from 2016 to 2022 (Figure 10). The first image was considered to show the daily sea state (daily refers to the absence of flooding or storm surge events, [5] (Figure 10a). The other images display an evident plume diverted northward (red line in Figure 10) by the dominant wave direction (220°–260°) at the *Arno River, Serchio River*, and *Scolmatore channel* mouths and a larger area affected by suspended sediment (green-light blue colours) on 26 and 28 February 2020 (Figure 10e,f) when the significant wave heights increased.

In Figure 10b,d a reduced sediment dispersion is evident due to a quieter sea state (Hs ≤ 1 m, SIR) than in the other images. On 3 March 2017 (Figure 10c), the Hs is about 1 m, but during the previous days, this parameter reached 7 m (SIR). Similarly, on 26 January 2021 (Figure 10h), the Hs was about 1 m, but on the previous day, the Hs was about 5 m. On 8 March 2018 (Figure 10d), the Hs varied between 2 and 3 m (SIR).

Furthermore, the last three images in Figure 10 show an overview of the study area during the time window investigated in the present work. In detail, the images dated 26 and 28 February show an increase in the sediment dispersion phenomenon during the storm surge, and the image of 4 March 2020 (Figure 10g) shows three clearly visible plumes at the *Arno River*, *Serchio River* and *Scolmatore channel* mouths, caused by the flooding event that occurred on 3 March 2020.

## 5. Discussion and Conclusions

This research was focused on the role of the Wave Radar Mobile System in the study of sea state and seabed depth changes in coastal areas during storm surges. The mouth of the *Arno River* was chosen as the study area for both its exposure to storm surges and the intense urbanization of the coastal zones. The importance of studying coastal areas by integrating data from different monitoring stations and through observation systems operating at different temporal and spatial scales was also emphasised.

During storm surges, radar images showed areas where sediments were prone to settle, forming new bottom shapes (shoals, bar structures), locally varying the depth of the seabed. The latter are probably due to both storm surges, coming from 220°–260°, which eroded the sediments from the seabed and moved them landwards, and to the longshore current, which moved the sediments mainly northwards. This direction is clearly evident in the Sentinel 2 images showing the northward deviation of the plumes of the *Arno River*, *Serchio River*, and the *Scolmatore channel* over several years. Furthermore, the seabed shape, a fan-delta-like structure with a depth of 6 m, in the radar image of 3 March 2020, has grown during the concurrence of a storm surge and flooding events. The interaction between the river flow, rich in sediment from inland and eroded from the river bed, and sea waves, rich in sediment eroded from the seabed, favoured the sedimentation process. The accuracy of the seabed depth map (RMS ± 0.77 m) was validated with single-beam measurements made immediately after a storm surge.

The data acquired using the X-band radar technology demonstrate that the Remocean Radar Mobile System is a valuable monitoring tool that improves the knowledge of the relations between sea conditions, nearshore sediment dynamics, and seabed changes at a local scale. The ease of its use and user-friendliness is efficient for effective analyses of rapid coastal changes. The accuracy of traditional oceanographic instruments is essential; the coastal radar system does not overcome this challenge but supports their use. Another aspect shown in the results concerns the comparison of the measurements made by the different instruments—the echosounder and the radar system. As soon as the radar beam moved away from the land, and especially as the coastal depth increased, an area was reached where the accuracy of the coastal radar decreased substantially for objective reasons, but the possibility of using coastal radar for the analysis and monitoring of coastal bathymetry showed its own usefulness during the storm event, when it was impossible to go out with a boat for a survey. In addition, the X-band radar data recorded over many years and appropriately organized into a database will be a fundamental source for integrated coastal zone management, the goals of which are to prevent and reduce the impact of hazardous events and to promote sustainable development. The maintenance of natural resources and environmental quality help to guarantee future generations the same ecosystem services we enjoy today. Future developments will concern the possibility of analysing monitoring data over a longer period of time and for many mouths in order to better understand the radar system’s ability to detect seabed changes related to interactions of river flow, flooding and wave climate.

## Figures and Tables

**Table 1:** Significant wave height and flow rate values for both studied and storm surge events visible from the available Sentinel 2 images are shown. The symbol “–” indicates the lack of a storm surge event.

Date	Hs (m)	Flow Rate (mc/s)	Note
8 December 2016	-	-	-
26 February 2017	<1	421	Hs = 2 m on 25 February; flow rate = 800 mc/s on 25 February
8 March 2017	1	455	Hs = 5 m from 5 to 7 March flow rate = 756 mc/s on 7 March
8 March 2018	2.3	556	flow rate = 567–993 mc/s from 7 to 3 March
26 February 2020	5.5	48	
28 February 2020	4	124	
4 March 2020	3	592	Hs = 3 m for several day before 4 March; flow rate= 897 mc/s on 3 March
26 January 2021	1	622	Hs = 5.3 m from 22 to 25 January

## Data Availability

Sentinel 2 images are available at https://sentinels.copernicus.eu/web/sentinel/sentinel-data-access, accessed on 30 July 2022; buoy data are available at https://www.sir.toscana.it/mareografia-pub; hydrometric data are available at https://www.sir.toscana.it/idrometria-pub; Wave Radar data are available upon request to francesco.raffa@igg.cnr.it.
